# *Ex-vivo* Smear Layer Removal Efficacy of Two Activated Irrigation Techniques After Reciprocating Instrumentation in Curved Canals

**DOI:** 10.2174/1874210601711010512

**Published:** 2017-10-16

**Authors:** Tamara Costa Lopes Schiavotelo, Marcelo Santos Coelho, Luis Cardoso Rasquin, Daniel Guimarães Pedro Rocha, Carlos Eduardo Fontana, Carlos Eduardo da Silveira Bueno

**Affiliations:** 1 Department of Endodontics, School of Dentistry and Medicine, São Leopoldo Mandic University, Campinas, SP, Brazil; 2 Department of Integrated Clinical Dentistry and Diagnostics, School of Dentistry, Federal University of Bahia, Salvador, BA, Brazil; 3 Department of Endodontics, School of Dentistry and Medicine, Pontificia Universidade Católica de Campinas, Campinas, SP, Brazil

**Keywords:** EndoActivator system, Root canal, Smear layer, Sonic irrigation, Ultrasonic irrigation, Distobuccal roots

## Abstract

**Introduction:**

This study aimed to compare the effectiveness of two activated irrigation techniques in removing the smear layer after single-file reciprocating instrumentation in curved canals.

**Materials and Methods:**

Sixty distobuccal roots of maxillary molars were standardized to create a closed system, and then instrumented using WaveOne Primary (Dentsply Maillefer, Ballaigues, Switzerland) instruments. Fifty-four specimens were randomly distributed into 3 groups for final irrigation: Non-activated irrigation, passive ultrasonic irrigation (PUI), and EndoActivator (EA;Dentsply Maillefer, Tulsa, USA) irrigation. All specimens received 3 mL of 17% EDTA for 1 minute, followed by irrigation with 6 mL of 2.5% NaOCl. The apical, middle and cervical thirds of the specimens were analyzed using scanning electronic microscopy (SEM), and the amount of remaining smear layer on the canal walls was rated by three examiners using a five-category scoring system. Kendall’s concordance coefficient was used to assess inter-rater agreement. Kruskal-Wallis and Mann-Whitney (Bonferroni) tests were used to compare the scores.

**Results:**

Kendall’s concordance coefficient was ≥ 0.7, indicating an excellent level of agreement between the raters. No statistically significant difference in irrigation techniques efficacy for removal of the smear layer (*p*=0.061) was found for the apical third. The scores attributed to the specimens irrigated with the EA system were significantly lower than those of the other groups in the cervical and middle thirds (*p*< 0.05).

**Conclusions:**

The efficacy of the EA system in removing the smear layer in the cervical and middle thirds of root canals instrumented with reciprocating motion was significantly higher than that of either PUI or non-activated irrigation. Both EA and PUI performed similarly in apical third.

## 1 INTRODUCTION

Root canal preparation procedures have become quicker to perform with the more recent automated instrumentation and contemporary endodontic instruments and devices [1]. However, endodontic instruments are unable to prepare the entire surface of irregular root canals, leaving 30% to 50% of the root canal system uninstrumented [2]. The smear layer is a muddy, non-homogeneous material produced during instrumentation, which adheres only weakly to the root canal walls. It is composed of organic and inorganic particulates, coagulated proteins, pulp material and blood cells, and also harbors bacteria and fungi from infected canals [2, 3].

One of the main goals of endodontic irrigating procedures is to remove residual biofilm from non-instrumented surfaces and to remove the smear layer created on the instrumented surfaces [3, 4]. The complete removal of the smear layer is extremely important to promote direct contact of the irrigation solution with the root canal wall, provide adequate disinfection and avoid the presence of filling debris that can increase the probability of bacterial infiltration in the root canal wall–filling material interface [3, 5].

Ultrasonic agitation has been reported in the literature as an effective active irrigation option [6, 7]. Compared with non-activation, activation of a sodium hypochlorite (NaOCl) solution can dissolve tissue and disrupt bacteria more effectively, whereas activation of ethylenediaminetetraacetic acid (EDTA) in the final phase of irrigation has proven to remove the smear layer more effectively [2, 7].

The EndoActivator (EA; Dentsply Tulsa, Tulsa, OK, USA) system is used in endodontic treatment to apply sonic energy to the irrigation procedure. The cavitation and acoustic “streaming” produced by the system improve debridement and cause disorganization of the smear layer and biofilm. The fluids thus activated promote thorough cleaning and disinfection of the root canal system [8].

The ability of single-file reciprocating instruments to remove inorganic debris has been shown to be uncertain, and instrumentation protocols are still needed to reduce debris buildup, particularly in view of the compactness of the debris produced during reciprocating motion in canals with a high prevalence of isthmuses and protrusions [9].

PUI and the irrigation produced with the EA system have been compared in respect to smear layer removal and debris elimination effectiveness after rotary instrumentation [10, 11]. The treatments conducted have shown that PUI and the EA system produce comparable smear layer removal results when used with rotary instrumentation [10, 11]. To our knowledge, however, few studies have been conducted to compare both irrigation protocols in this respect, after single-file reciprocating instrumentation [12].

Thus, the objective of this study was to compare the effectiveness of one non-activated and two activated irrigation techniques in removing the smear layer after single-file reciprocating instrumentation in curved root canals. The null hypothesis was that there would be no differences among these three irrigation techniques in regard to smear layer removal.

## 2 MATERIALS AND METHODS

### 2.1 Sample Size Calculation

The total sample size for this study was calculated using G*Power 3.1 for Windows software (Heinrich-Heine-Universität, Düsseldorf, Germany). The minimum sample size required per group was 13, considering a significance level of 5%, a power of 95%, and estimating the root canal cleaning effect promoted by activated irrigation techniques, compared with that of a non-activated method.

### 2.2 Specimen Preparation

This study was approved by the local research ethics committee (protocol no. 2012/0153). Sixty human maxillary molars extracted for periodontal reasons were selected for the study. They had no obvious anatomical changes, and curved canals with angles between 20 and 40 degrees. The teeth were washed with water and stored for one week in a 0.1% thymol solution at room temperature until they were used in the experiments. An initial selection of 60 teeth was made to ensure a minimum of 13 specimens for each of the 3 groups after the preparation process.

The specimens were decoronated with diamond discs and their distobuccal roots were standardized to a length of 10 mm. Foraminal patency was confirmed with a stainless steel #10 K-file (Dentsply Maillefer). The working length was established by subtracting 1 mm from the length determined after the tip of the file was observed at the apical foramen using a laboratory light microscope (Stemi DV4 spot; Carl Zeiss, Oberkochen, Germany) under 20X magnification.

Each tooth was radiographed in the buccolingual and mesiodistal directions, with a #10 K-file inserted up to the working length to reveal root canal anatomy and confirm a root canal curvature higher than 20º and lower than 40º, according to the method described by Schneider [13].

The apexes of the roots were occluded with wax pellets and then embedded in plastic cylinders filled with polyvinyl siloxane impression material in order to create a closed system to simulate the periodontal ligament and prevent extrusion of debris during preparation, following the method modified from that used by Tay *et al.* [14]. Eighteen specimens per group (54 in all) were considered viable for the experiment at the end of the preparation process. Two specimens in each group were lost during the preparation and were discarded.

### 2.3 Root Canal Instrumentation

All specimens were instrumented by a single endodontic specialist (TCLS) using the WaveOne Primary (25/08) instrument (Dentsply Maillefer) powered by an X-Smart Plus electric motor (Dentsply Maillefer) in reciprocal motion up to the working length, according to the manufacturer’s instructions. Each instrument was used in 4 specimens and then discarded [15]. The canals were irrigated with 3 mL of 2.5% NaOCl, before the instrument was first inserted and after insertion in each of the root canal thirds (cervical, middle and apical), for a total of 4 insertions and 12mL of irrigating solution. The solution was injected with a disposable 5 mL syringe and 30G irrigation tip (NaviTips; Ultradent, South Jordan, UT, USA). In addition, apical patency was confirmed with #10 K-file after performing each irrigation step.

The specimens were randomly assigned (http://www.random.org) to one non-activated and two activated irrigation groups of 18 roots each for the final irrigation [16, 17], as follows:

Group 1 (n = 18): Non-activated irrigation with 3 mL of 17% EDTA for 1 minute without agitation, followed by irrigation with 6 mL of 2.5% NaOCl.

Group 2 (n = 18): PUI with 3 mL of 17% EDTA for 1 minute followed by irrigation with 6 mL of 2.5% NaOCl. Each solution was activated for 3 cycles of 20 seconds (13). A size 25.01 Irrisafe tip (Satelec Acteon; VDW, Munich, Germany) attached to a P5 Newtron XS ultrasonic device (Satelec Acteon, Merignac-Cedex, France) was used to perform PUI at a power setting of 10 and a distance of 2 mm short of the WL, as recommended by the manufacturer.

Group 3 (n = 18): EndoActivator agitation of 3 mL of 17% EDTA for 1 minute followed by 6 mL of 2.5% NaOCl. Each solution was activated for 3 cycles of 20 seconds. The red, medium-size tip (25/04) of the EndoActivator system was placed 2 mm short of the working length, and set to operate at 10,000 cpm, as recommended by the manufacturer.

Immediately after the final irrigation, the root canals in all the groups were dried with a 0.14 capillary tip (Ultradent, South Jordan, UT) and WaveOne Primary paper points.

### 2.4 Tooth Sectioning and Preparation for SEM

Two longitudinal grooves were made on the buccal and lingual surfaces of the roots using a diamond disk, without entering the root canal space, to facilitate cleavage of the roots into two halves [16]. Each specimen was dehydrated in ethanol, dried, and coated with gold, to allow the visualization of the internal walls of the root canals with a scanning electron microscope (SEM; JSM 6390LV, JEOL, Tokyo, Japan).

**Fig. (1):**
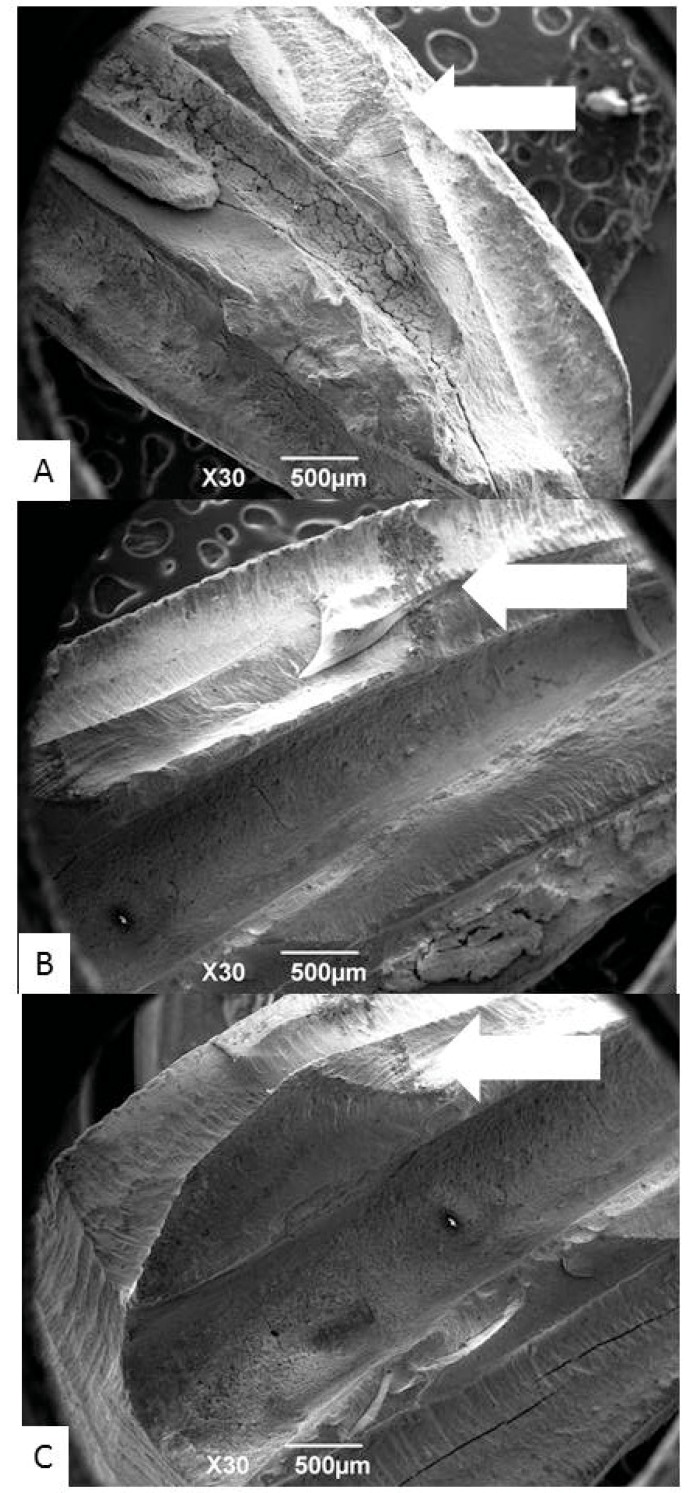
Marks done with graphite to determine ROIs (arrows) (A) 2mm-apical, (B) 5mm-middle, (C) 8mm-cervical SEM view 30X.

The cleaved specimens were marked with graphite lead at the center of the cervical, middle and apical thirds, respectively 8, 5 and 2mm from the apical foramen (Fig. **1**).

### 2.5 Scanning Electronic Microscopy Evaluation

Initially, 14X magnification was used for overall visualization of the areas on the specimens marked for observation. Then, 1000X magnification was used to assess smear layer removal, by taking a digital image and attributing a score to each third. A total number of 162 images were obtained. The images were then blindly evaluated by three examiners, with experience in research in Endodontics, that were not involved in the preparation of the specimens. The median value of three examiners was taken as the rate of each evaluation.

The remaining amount of smear layer on the canal walls was rated according to a five-category scoring system, modified from Hülsmann *et al.* [18], and Schäfer & Lohmann [19], as follows: Score 0, completely clean surface with all dentinal tubules open; Score 1, smear layer covering less than 50% of canal walls, and most dentinal tubules open; Score 2, surface covered by a thin smear layer with roughly half of the dentinal tubules open; Score 3, smear layer covering more than 50% of canal walls, and few open dentinal tubules; Score 4, surface entirely covered by smear layer with no open dentinal tubules.

## 3 STATISTICAL ANALYSIS

Kendall’s concordance coefficient was used to assess inter-examiner agreement. After homogeneity testing the non-parametric results were tested by the Kruskal-Wallis and Mann-Whitney (adjusted by Bonferroni correction) tests were used to measure and compare the smear layer removal results. All the analyses were performed using Minitab software (Minitab Inc., Chicago, IL, USA). The level of significance adopted was 5%.

## 4 RESULTS

Kendall’s concordance coefficient was calculated at ≥ 0.7, indicating an excellent level of agreement between the evaluators.

No statistically significant difference in smear layer removal was found between the irrigation techniques tested in the apical region (*p* = 0.061). In this region, most specimens were attributed scores of 3 and 4.

The EndoActivator specimens were attributed the lowest scores for the cervical and the middle thirds. These scores were significantly lower than those attributed to the PUI specimens.

The remaining smear layer scores for all regions are summarized in (Table **1**).

**Fig. (2):**
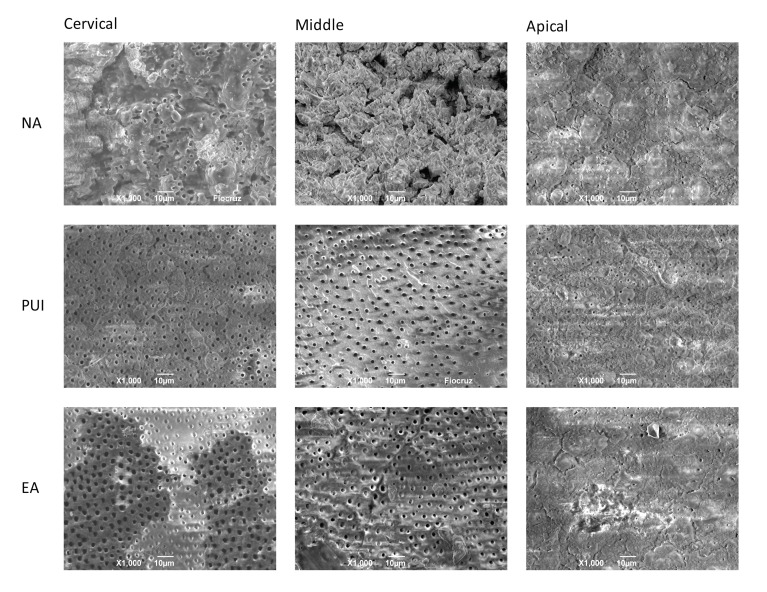
Representative SEM images of smear layer removal from different root thirds, after applying the irrigation techniques being tested. NA: Non-activated; PUI: Passive ultrasonic irrigation; EA: EndoActivator system.

All the groups showed increasingly better smear layer removal results as the analysis moved from the apical to the cervical region (Fig. **2**).

## 5 DISCUSSION

The objective of this research was to compare the effectiveness of one non-activated and two activated irrigation techniques, in removing the smear layer after single-file reciprocating instrumentation in curved root canals. Based on the statistical analysis of the results, the null hypothesis of the study was rejected for the middle and cervical thirds, insofar as the amount of smear layer remaining on these root canal walls was not the same for the different study groups.

Only curved root canals were selected for this study because they are more difficult to instrument, irrigate, and clean than straight canals [11, 20-22]. An *in vitro* closed-end canal model can more accurately simulate *in vivo* conditions, such as gas entrapment in the root canal and periodontal ligament. It can also avoid overflow of the irrigating solution [10, 11, 14]. Accordingly, in the present study, the apices of the roots were covered with a small amount of wax and placed in plastic cylinders filled with silicone to produce a closed-end system.

Several studies have reported that a NaOCl solution is recommended as the main irrigant, owing to its ability to dissolve pulp tissue and its efficiency on biofilm. A 2.5% NaOCl solution was chosen in the present study, in accordance with a previous study employing a similar methodology [11]. As an auxiliary solution for smear layer removal, 17% EDTA was observed in previous studies [23, 24]. In this study, the amount of irrigant was standardized at 3 mL of 17% EDTA and 6 mL of 2.5% NaOCl, activated for 3 cycles of 20 seconds each. These conditions have proven more effective in removing debris during ultrasonic vibration [24].

This study used a five category scoring system to rank the remaining smear layer of the walls of the roots in a similar manner as done in previous studies [10, 21, 22]. Other studies, however, used 3-category scoring [11] or 4-category scoring [25, 26].

Overall, the results of the present study showed better removal of smear layer at coronal and middle levels than at apical third, corroborating previous findings [10, 21, 27].

In the present study, EA system performed better than PUI in coronal and middle third with regard to smear layer removal, corroborating the findings of previous studies [21, 22]. Mancini *et al.* [10] also presented better results for EA at 5mm and 8mm from the apex - the same distances adopted in the present study. The superiority of EA in middle thirds can be attributed to the flexibility of its tip, which can easily follow the canal curvature, whereas the rigid ultrasonic tip cannot. Yet, the size of EA (25.04), closer to the final preparation used (25.08), might permit the removal of smear layer during activation without producing additional debris. One may claim that, at coronal level, PUI activation might be limited whether the ultrasonic tip touches the root canal walls or not.

Using a final preparation of 40.02, Rödig *et al.* [21] showed no difference among EA, PUI and non-activated group in smear layer removal at apical third. However, other studies [11, 22], using smaller preparations, found both EA and PUI irrigation techniques superior to no-activation techniques in dentinal tubule cleaning at apical third. The different apical sizes used in the final preparation of the specimens are a possible reason for the conflicting results in those previous studies. The preparation size used in our study is similar to the ones used by Caron *et al.* and Blank-Gonçalves *et al.* [11, 22], but the kinematics used in our study were different and our results are in disagreement with those findings. Robinson *et al.* [9] have demonstrated that reciprocating kinematics led to more debris remaining than rotary kinematics. We can hypothesize that the reciprocating instrumentation used in the present study has led to higher amounts of smear layer than the rotary systems used by Caron *et al.* and Blank *et al.*, thus resulting in its limited removal.

While in the present study EA and PUI performed similarly at apical level, Kato *et al.* [26] found better results for EasyClean than for PUI at apical levels. The EasyClean evaluated by Kato *et al.* can be compared to EA, as it is a flexible plastic device presenting same tip and taper (25.04) as EA. However the reciprocating kinematics used in that device might have improved the results at apical third while EA and PUI performed similarly at this level.

The limitations of the present study are clear: SEM does not provide 3D visualization. Additionally, regions of interest (ROI) may be difficult to establish precisely. This limitation was addressed by using a graphite lead to demarcate ROIs, in addition to low magnification rates. Moreover, results from an *in vitro* study should be carefully evaluated prior to establishing clinical protocols. Therefore, clinical studies in this topic are recommended.

## CONCLUSION

Within the limitations of the present study EndoActivator showed the best performance in removing the smear layer from root canal walls in the cervical and middle root thirds. No statistically significant difference among the three irrigation techniques was observed for the apical region.

## Figures and Tables

**Table 1:** Mean value (SD) of scores in the 3 different regions according to the activation system used.

Distribution of Scores per Region			
	EndoActivator	Ultrasound	Non-Activated
Mean	Mean	Mean
Apical	3.00 (1.33)^a^	3.83 (0.38)^a^	3.89 (0.32)^a^
Middle	1.61 (1.42)^b^	2,78 (1.40)^c^	3.50 (1.04)^c^
Cervical	1.50 (1.15)^b^	2.67 (1.46)^c^	3.39 (0.70)^c^

*Values with different superscript letters were statistically different at *P* = .05.
